# A deep learning based approach for automated plant disease classification using vision transformer

**DOI:** 10.1038/s41598-022-15163-0

**Published:** 2022-07-07

**Authors:** Yasamin Borhani, Javad Khoramdel, Esmaeil Najafi

**Affiliations:** 1 Center of Excellence in Robotics and Control, Advanced Robotics & Automated Systems (ARAS), Faculty of Mechanical Engineering, K. N. Toosi University of Technology, Tehran, Iran; 2 Faculty of Mechanical Engineering, Tarbiat Modares University, Tehran, Iran

**Keywords:** Pattern recognition receptors in plants, Patterning, Plant immunity, Electrical and electronic engineering

## Abstract

Plant disease can diminish a considerable portion of the agricultural products on each farm. The main goal of this work is to provide visual information for the farmers to enable them to take the necessary preventive measures. A lightweight deep learning approach is proposed based on the Vision Transformer (ViT) for real-time automated plant disease classification. In addition to the ViT, the classical convolutional neural network (CNN) methods and the combination of CNN and ViT have been implemented for the plant disease classification. The models have been trained and evaluated on multiple datasets. Based on the comparison between the obtained results, it is concluded that although attention blocks increase the accuracy, they decelerate the prediction. Combining attention blocks with CNN blocks can compensate for the speed.

## Introduction

Early diagnosis of plant disease is essential for producing healthy products. As humans use the different kinds of plants to provide food, it is necessary to reduce the harmful effects of plants disease. In some cases, farmers can not diagnosis the symptoms of plant illness accurately because of the tiny features. Moreover, a number of farmers are not expert in diagnosing the disease, so that the artificial intelligence can help them to provide a better recognition. In the past years, convolutional neural networks have been chosen for image processing as they have progressed in these fields. Recently the Vision Transformer (ViT) structure has been introduced to improve the classification applications. The idea is based on how humans classify images. When a human observes a picture, he or she focuses on a specific area of the image to detect the instance of interest. The ViT structure follows this approach for image classification.

It is prevalent to use pre-designed architectures like ResNet, ViT, etc., for computer vision tasks. These models usually have lots of trainable parameters, requiring a large dataset to find the optimal values for their parameters. Hence, these models are usually trained on a massive dataset like ImageNet; then, the obtained weights are used for transfer learning. High accuracy will be acquired when the second dataset has a similar domain to the first dataset. Still, when the domain is different, the models would struggle to reach an acceptable accuracy on small datasets. In addition to that, the pre-designed architectures are usually heavy models with lots of computations which slowdowns the prediction. For real-time applications, the prediction needs to be as fast as possible. A question may arise, and an argument can be made whether it is always an excellent choice to go for transfer learning or whether light-weighted hand-designed architectures can achieve adequate performance?

### Related works

In the literature, studies have been conducted on diagnosing the diseases of a particular plant. Most of these datasets contains images that are captured in the acceptable circumstances since in the agriculture purposes the images are usually recorded in a good conditions. For example, for diagnosing wheat rust, aerial and non-aerial images of wheat farms were collected in^1^. The images were labeled for object detection. They proposed to localize the wheat leaf with object detection networks. After finding the corresponding bounding box, the cropped box is the input of a classification network to obtain the true class of that instance. Overall, five CNN models were trained for classification including VGG16^2^, ResNet-50^3^, Inception^4^, MobileNet-V3^5^ and EfficientNet-B0^6^. It was concluded that the EfficientNet-B0 has the highest accuracy while it has a lower computational cost.

Since there are many structures for machine learning algorithms, different kinds of models have been used like the C-DenseNet structure in^7^. Their dataset consists of 5 different categories, and it was considered as a balanced dataset because of the distribution of images in each class. Also, the level of the disease intensity is divided into six classes. In order to preprocess the images, all of them were cropped. Because of that, the background of the pictures was not complicated. As the image features in some levels have slight differences, the C-DenseNet was used in order to improve the results. In this structure, the convolutional layers were used. Between these convolutional layers, the dense layers were implemented. In addition to that, Convolutional Block Attention Modules (CBAM) were used which consist of the channel and spatial attention modules. The channel attention module uses fully connected layers to extract features from the feature map, then uses sigmoid to find which part of the input channel needs more attention. The outputs of these two modules are multiplied by the input feature map and the output of the CBAM is obtained. As the attention mechanism idea is used to build the model structure, the key features of images are detected more accurately. The accuracy of their study is about 97.99%.

In addition to the previous studies, the matrix-based convolutional neural network M-bCNN structure^8^ was implemented. Because of the slight and tiny wheat leaf disease features, the matrix-based architecture was proposed in order to signify the important properties of images. The function of this proposed architecture suppress the AlexNet^9^ and VGG-16^2^ networks.

Another example of focusing on the disease of a specific plant can be found in^10^, where a few pictures of infected rice leaves were collected. The images were taken on a farm in India. To achieve a rice disease classifier, they first preprocessed images and removed the background from the complicated images. After that, a few simple image segmentation techniques like K-means clustering and Ostu’s method were applied to find the infected area on the leaves. The segmented images were used as the input to an SVM classifier to find the proper label for the image^10^.

Some datasets include multiple plants and their diseases, like the Plant Village dataset^11^. The original images are colored images (RGB). The grayscale version and segmented version of these images are also available.All the experiments in^12^ had been done on each of these versions separately. Several trainings with different configurations had been reported. AlexNet^9^ and GoogLeNet^13^ were used as the based models. Each version of dataset had been used for training different train-test splits ( (Train 20% , Test 80%), (Train 40%, Test 60%), (Train 60%, Test 40 %), (Train 80 %, Test %20) ). Each training was conducted once with the initial random weights (training from scratch) once with the transfer learning by using the weights of AlexNet and GoogLeNet, which were trained on the ImageNet; as the initial weights. As was expected, the models with transfer learning gained better performance as compared to the model trained from scratch.

In some papers, the main focus has been on specific class or classes from the Plant Village. For example, in^14^, the authors only focused on the tomato leaf images from the Plant Village and implemented a simple CNN for classifying the disease in the tomato leaves images. They used LVQ as the classifier of their network and reported an average accuracy of 86% on the test data. In^15^ also the tomato leaf images were considered. Instead of implementing a CNN from scratch, they used AlexNet and VGG16 architectures. Rather than using the raw RGB images, a segmented version of the images was used in which the value of the background pixels is set to zero. The accuracy of 97.49% is this paper. Although the segmented images simplify the classification task for the neural network, but in the real scenarios in the field, no segmented images are available, and it requires either humans to segment the images which is time-consuming or a neural network for segmentation. If there is a neural network for segmentation, that network can also perform the classification task. Moreover, a convolutional neural network for classification is capable of handling images with non-zero backgrounds by itself. By considering the same class from the Plant Village, Halil et al. tried to achieve a model which can be used in real-time applications. They trained AlexNet and SqueezeNet^16^ on the tomato leaves images which obtained the accuracy of 95.6% and %94.3 respectively. On Jetson Tx1, they reported the prediction time of 150 ms for AlexNet and 50 ms for SqueezeNet^17^.

The ViT idea has been used in agricultural applications in recent works like^18^ and^19^, which used the main versions of ViT (ViT-B16 with 16 and ViT-B32 with 32 attention blocks) without any changes to do the classification, or^20^, which utilized two ViT models in parallel to handle images with two different resolutions. Some of these works focused on the disease of one specific plant^19,20^, and a some of them focused on the classification of plants and not their diseases^18^. However, when the number of plants and diseases increases, the problem becomes more challenging. Moreover, the prediction speed was not studied, which is crucial for real-time classification. Employing such a heavy network for plant disease classification might be extreme, and shallower models might also have sufficient performance in some situations.

This paper introduces lightweight models for real-time crop disease classification using ViT structure. The proposed models will be challenged on three different datasets with a different number of images. With respect to accuracy and prediction speed, these models are compared with CNN-based architecture with almost the same complexity. Combinations of CNN and ViT are also investigated for the possibility of improving the results. Furthermore, the effect of image size on the results will be discussed.

## Background

Based on convolutional neural networks and ViT, the networks utilized in this paper were constructed. Accordingly, in this section these main structures are briefly explained.

### Convolutional neural networks

The convolutional neural network was introduced by Yen leCun in the 1980s^21^. In 2012, when AlexNet^9^ won the challenge of ImageNet, the CNNs have become more popular, and they have been used in different projects for image classification and recognition, processing languages, medical images, etc. In the Convolutional Neural Networks (CNN) structure, the image features are extracted with the preservation of the 2D structure, while this structure consists of a number of filters. By sliding the filters over the input image, the calculation for extracting features takes place.

### Vision transformer

The Vision Transformer (ViT)^22^ is a novel idea for training neural networks on the images. As they presented, this structure can reach higher accuracy in comparison with ResNet152^3^ on some classification datasets like ImageNet^23^, CIFAR-100^24^, Oxford-IIIT Pets^25^ and outperforms the Noisy Students (EfficientNet-L2)^26^.

ViT is inspired by Bert^27^ and attention is all you need^28^ papers which take advantage of the attention mechanism for Natural Language Processing (NLP) applications and introduced the concept of the transformer. The transformer structure gets the sequence of the 1D input array. For processing 2D images, at first 2D patches are extracted from them, then they are reshaped to create 1D arrays, hence are appropriate for ViT structure. In order to complete preparing the patch embedding for the next layer, they are added to the positional encoder. The positional encoder helps the network to remember the relative position of the patches with respect to each other. In the next step, the inputs are normalized with the normalization layer^29^, then they enter the transformer block. The most important part of this block is the multi-head attention layer. The purpose of the multi-head attention layer is to calculate weights to allocate higher values to the more important areas. In other words, it concentrates the network attention on the more essential parts. The output of the multi-head attention layer is a linear combination of each head.

## Implemented datasets

In this paper, three different datasets are used in order to evaluate the above-mentioned models. The first is a gathered small dataset which we call it *Wheat Rust Classification* dataset, the second is *Rice Leaf Disease* dataset, and the third is the popular dataset named *Plant Village*.

### Wheat rust classification dataset

**Figure 1:**
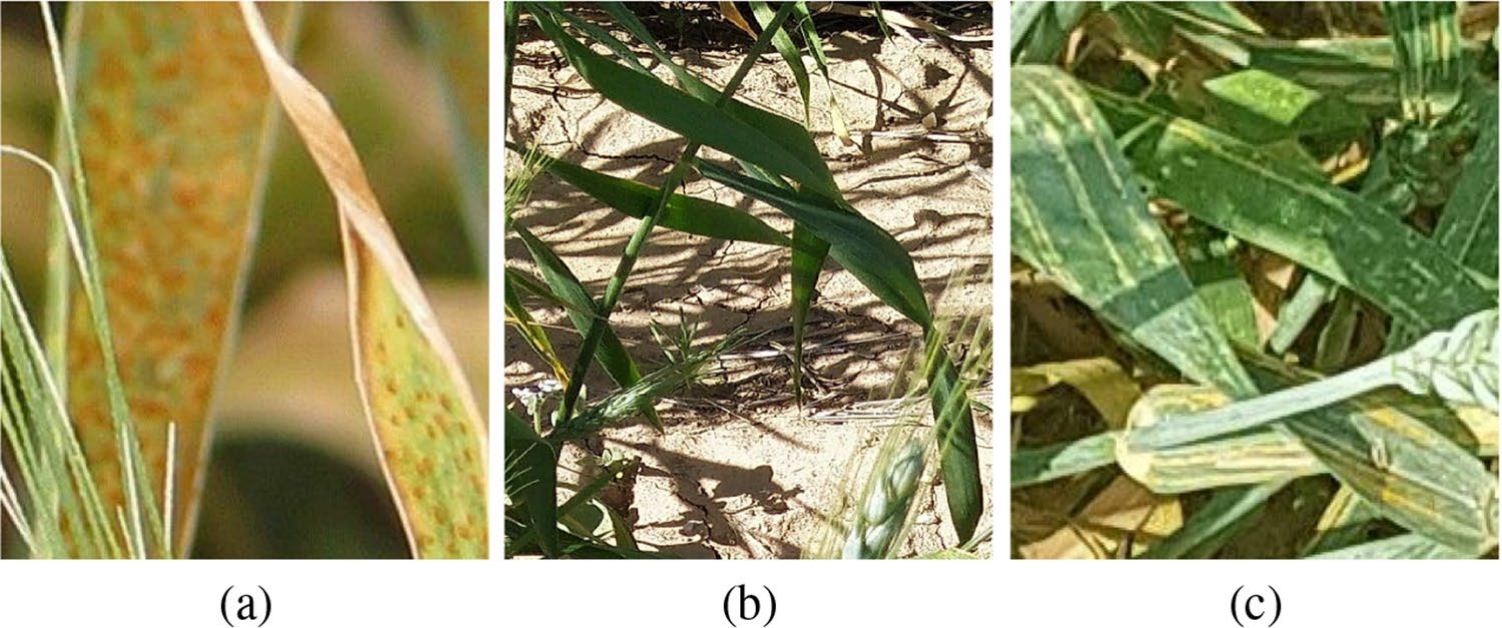
Sample images from Wheat Rust Classification Dataset: (**a**) brown rust, (**b**) healthy wheat, and (**c**) yellow rust.

The first dataset used in this paper is collected by Safari et al.^1^, which is called “Wheat Rust Classification Dataset (WRCD)”. The Wheat Rust Classification dataset includes three classes: healthy wheat, yellow rust and brown rust. As shown in Figure 1, the yellow rust class includes yellow dotes on wheat and brown rust makes wheat suffer from brown dotes. The other difference between them is how dots are aligned in these two categories. In the yellow rust class, the dots are approximately aligned in a row, but the brown rust dots does not have a particular pattern. So these structures can be helpful for distinguishing the brown and the yellow rust. The healthy class shows wheat without infections.

In this dataset, the Brown Rust class has 1128, the Yellow Rust class has 1156 and the Healthy class has 1395 pictures. Hence, the samples are approximately distributed uniformly in these three classes. The Wheat Rust Classification Dataset is available at: https://www.kaggle.com/sinadunk23/behzad-safari-jalal.

### Rice leaf disease dataset

**Figure 2:**
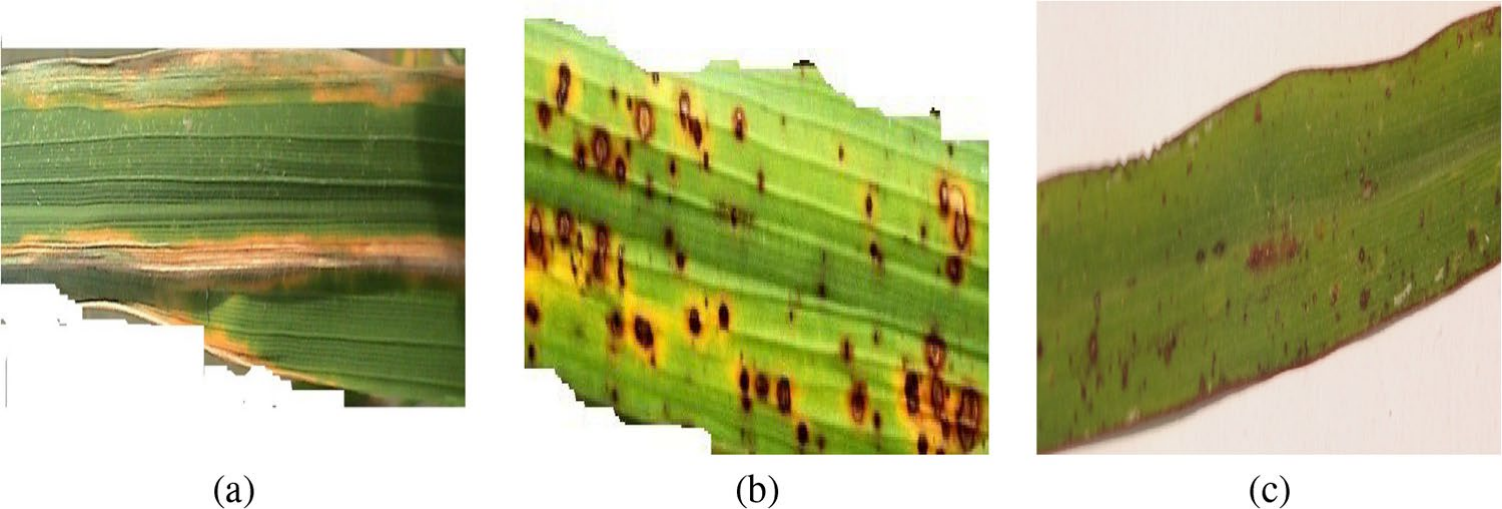
Sample images from Rice Leaf Disease Dataset: (**a**) bacterial leaf blight, (**b**) brown spot, and (**c**) leaf smut.

Rice Leaf Disease Dataset (RLDD) is a small database with only 120 images of infected rice leaves. These images have been taken from a rice field in India. There are three classes in this dataset: 1-Bacterial Leaf Disease, 2-Brown Spot and 3-Leaf Smut. Each class has 40 samples. Three random samples from the dataset are selected and shown in Figure 2. The background of these images are either removed and replaced with white color, as illustrated in Figure 2a,b, respectively) or it is very simple as depicted in Figure 2c. The Bacterial Leaf Blight symptom appears in yellow color and is elongated on the leaf. The Brown Spot and Leaf Smut are pretty similar to each other, but still, they differ from each other. The Brown Spot causes the circles with the bigger area on the leaves and the color of these circles is darker compared to the Leaf Smut. In the Leaf Smut infection, small dots are scattered through the leaf.

In this dataset, the main challenge arises from the limited number of images. In addition to that, Brown Spot and Leaf Smut symptoms are very similar to each other which makes the task even harder.

### Plant village

Plant Village is a large-scale dataset that contains 54,306 images of crop leaves. These leaves belong to 14 crop species which some of them are healthy and some of them suffer from the disease. 26 kind of different infection has been labeled. Here, the task is to identify the true class of the leaf (the crop species and also its disease) based on the input image. Overall, there are 38 classes in this dataset.

These images are taken in particular conditions which may differ from the real world scenarios (for example, the background has a simple texture and different color from the leaves). The distribution of images and these classes are not uniform; some classes have more than 5000 images while others have less than 200 samples. This imbalanced distribution makes the classification task more challenging.

## Network structure

In this paper, a number of structures which are described as follows are studied. At first, two main building blocks are defined as CNN and Transformer block:

### Convolutional neural networks block

**Figure 3:**
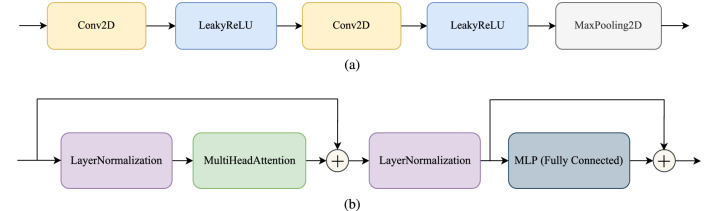
The schematics of the (**a**) convolutional block structure and (**b**) transformer block structure.

The CNN block consists of two convolutional layers with 3 by 3 kernels. In these two layers, padding and activation are not applied. The output of the second convolutional layer is entered into a leaky ReLU layer. Then one max pooling layer with 2 by 2 kernel is considered which can be seen in Figure 3a. The idea of using two consecutive convolutional layers with 3 by 3 kernels comes from the VGG structure which suggested using the smaller filters on top of each other has the same receptive field with bigger filters while they have less trainable parameters in comparison with the bigger ones^2^.

### Transformer block

The second block is the Transformer block. As it is shown in Figure 3b, the input of the block is fed to the layer normalization layer. The normalization layer is followed by a multi-head attention layer. Four attention heads are used in all the transformer blocks and the projection dimension is 64. Then the output of the multi-head attention layer adds to the input of the block with a skip connection. In the next step, the layer normalization layer is applied again, and the output of this layer goes to the fully connected layer. The final output of the block is calculated by summing the output of the fully connected layers and the input of those layers with another skip connection. It should be noted that in the mlp part shown in the block diagrams, two fully connected layers with 128 and 64 neurons exist and the activation of each of them is gelu, refer to^30^.

### Structure of models

**Figure 4:**
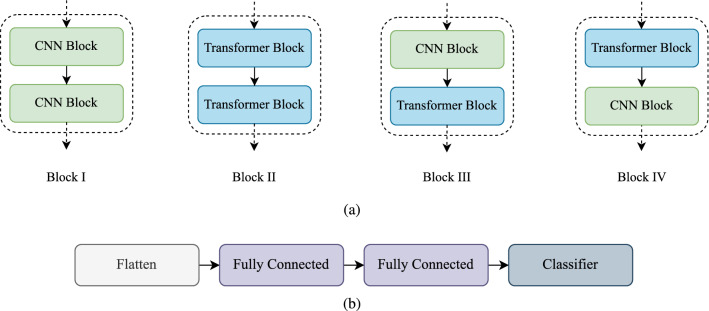
The main parts of models’ structure: (**a**) the backbone block diagrams and **b** the classification head of models.

The main structure of different backbones and a common head of the implemented models are shown in Figure 4. The backbones of Model 1 and Model 2 contain Convolutional Blocks only, which is shown as Block I in Figure 4a. Model 1 has one Block I, while Mode 2 has two of it. Model 3 and Model 4 have only Transformer Blocks in their backbone, shown as Block II in Figure 4a. Model 3 contains one Block II, while Model 4 contains two of it. Models 5, 6, 7, and 8 are hybrid as they contain both Convolutional Block(s) and Transformer Block(s). The backbones of Model 5 and Model 7 are structured by Block III and Block IV (shown in Figure 4), respectively. In Model 6, at first, Block I is used, and after that, Block II is attached to the output of Block I. In Model 8, at first, Block II is used and then Block I is attached to the output of Block II. All models have a similar classification head, shown in Figure 4b.

**Figure 5:**
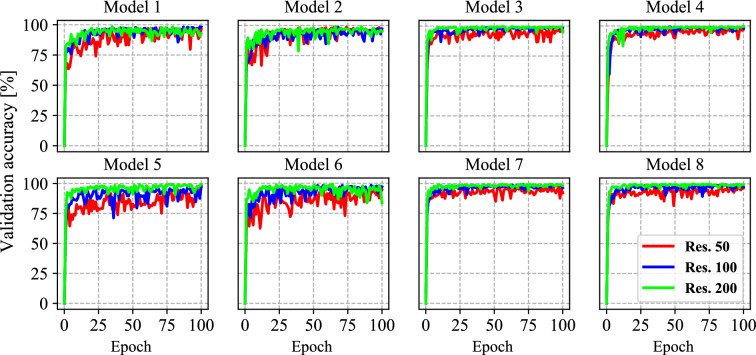
Accuracy of the models during training on validation set.

## Experimental results

In this section, the experiments on three datasets; Wheat Rust Classification, Rice Leaf Disease and Plant Village are explained.

### Experiment on wheat rust classification dataset

In this part, all the eight model structures have been used. Three different image resolutions were chosen for training the networks: 50 by 50, 100 by 100, and 200 by 200. The lower the input resolution, the faster the prediction speed, but the accuracy could decrease. Accordingly, the models were trained and evaluated starting from a low resolution (50 by 50). The resolution was doubled twice (100 by 100 and 200 by 200) to check the input resolution effect on the model’s accuracy and speed. The dataset has been divided into 80% of training and 20% of validation part. The optimizer used during training the models was the AdamW^31^. This optimizer can separately find the optimum learning rate and weight decay for changing these two parameters independently. All structures were trained for 100 epochs with an initial learning rate of 0.001.

#### Discussion

Figure 5 illustrates the accuracy of the models on the validation data during the training. The fluctuations in the charts are due to image augmentation, dropout layers, and data shuffling. It is worth noting that these oscillations almost have constant amplitudes, and there are no significant changes observed in the charts after the epoch 50. It may not be necessary to train these models for a more extended time, and 100 epochs seem sufficient to reduce the effect of randomness sources. The weights corresponding to the highest validation accuracy were stored and operated for the evaluation phase. It should be noted that in Figure 1a,c, the infected parts of the wheat leaves are occluded by other leaves and plants. However, the proposed model succeeded in gaining high accuracies on validation data.

The results of the evaluation are available in Table 1. All models are trained with three different resolutions. In order to validate the results, the average F1 score, recall, and precision are displayed. The Giga floating-point operations per second (GFlops) measurement is used to measure the computer’s performance. As this unit is decreased, the calculation cost will be reduced too. The convergence score is defined for comparing the results specifically, and it shows the integration of the validation accuracy per epochs. So the sooner the accuracy converges, the higher value this unit gets. Model 3, model 4, and model 8 with the input resolution of 200 by 200 have achieved the same average F1-score, recall, and precision with the EfficientNet model presented by^1^. However, these models have much lower computational costs based on GFlops in comparison with the EfficientNet. Among all these models, the model 3 and 4 have converged faster than others. Model 3 has the lower GFlops in comparison with Model 4. In the case of wheat rust classification, it seems that Model 3 with the resolution of 200 by 200 outperforms all the other models in terms of accuracy and GFlops.

**Table 1:** The results of the Wheat Rust Classification Dataset experiment.

Models	Res	Result of experiments				
		Average F1	Average recall	Average precision	GFlops	Convergence score
Model 1	50	0.97	0.96	0.97	0.006	0.88
	100	0.98	0.98	0.98	0.03	0.93
	200	0.98	0.98	0.99	0.12	0.93
Model 2	50	0.98	0.98	0.98	0.007	0.91
	100	0.97	0.97	0.97	0.032	0.91
	200	0.99	0.99	0.99	0.14	0.93
Model 3	50	0.98	0.98	0.98	0.003	0.96
	100	0.99	0.99	0.99	0.012	0.92
	**200**	**1**	**1**	**1**	**0.053**	**0.97**
Model 4	50	0.98	0.98	0.98	0.005	0.96
	100	0.98	0.99	0.99	0.02	0.92
	**200**	**1**	**1**	**1**	0.089	**0.97**
Model 5	50	0.97	0.96	0.97	0.006	0.83
	100	0.98	0.98	0.98	0.025	0.90
	200	0.99	0.99	0.99	0.1	0.95
Model 6	50	0.94	0.94	0.95	0.009	0.82
	100	0.98	0.97	0.98	0.039	0.95
	200	0.98	0.98	0.99	0.17	0.94
Model 7	50	0.97	0.97	0.97	0.002	0.92
	100	0.99	0.99	0.99	0.008	0.95
	200	0.99	0.99	0.99	0.037	0.96
Model 8	50	0.97	0.97	0.97	0.03	0.92
	100	0.99	0.99	0.99	0.012	0.95
	**200**	**1**	**1**	**1**	**0.053**	0.96
EfficientNetB0^1^	–	**1**	**1**	**1**	0.794	-

Significant values are in bold.

**Table 2:** The results of the Rice Leaf Disease Dataset experiment.

Models	Res	Result of experiments				
		Class	F1	Recall	Precision	Converg. score
Model 1	50	BLB	0.94	1.00	0.89	**0.69**
		BS	0.93	0.88	1.00	
		LS	0.88	0.88	0.88	
		Average	**0.917**	**0.920**	0.923	
Model 3	50	BLB	0.93	0.88	1.00	0.65
		BS	0.93	0.88	1.00	
		LS	0.89	1.00	0.80	
		Average	**0.917**	**0.920**	**0.930**	
Model 5	50	BLB	0.82	0.88	0.78	0.61
		BS	0.88	0.88	0.88	
		LS	0.80	0.75	0.86	
		Average	0.830	0.840	0.840	
Model 7	50	BLB	0.93	0.87	1.00	0.65
		BS	0.94	1.00	0.88	
		LS	0.88	0.88	0.88	
		Average	**0.917**	0.917	0.920	
Segmentation + SVM^10^	–	BLB	**1.00**	–	–	–
		BS	0.80	–	–	
		LS	0.40	–	–	
		Average	0.730	–	–	

Significant values are in bold.

**Table 3:** The results of the plant village experiment.

Models	Res	Result of experiments			
		Average F1	Average recall	Avarage precision	Convergence score
Model 2	200	0.9581	0.9586	0.9599	0.90
Model 4	200	0.9877	0.9877	0.9878	**0.95**
Model 6	200	0.9838	0.9837	0.9840	0.93
Model 8	200	0.9783	0.9783	0.9788	0.93
AlexNet^12^ (trained from scratch)	256	0.9782	0.9782	0.9782	–
GoogleNet^12^ (trained from scratch)	256	0.9837	0.9837	0.9837	–
AlexNet^12^ (transfer learning)	256	0.9928	0.9928	0.9927	–
GoogleNet^12^ (transfer learning)	**256**	**0.9935**	**0.9935**	**0.9935**	–

Significant values are in bold.

**Table 4:** The devices inspection used for measuring the prediction speed of the models.

Device	Accelerator	Specification
1	CPU	Intel(R) Xeon(R) CPU @ 2.20GHz with 1 core
2	CPU	Intel(R) Xeon(R) CPU @ 2.20GHz with 2 cores
3	GPU	Tesla K80
4	GPU	Tesla P4

### Experiment on rice leaf disease dataset

The Leaf Rice Disease Dataset has a few images. Since the complexity of the model should be chosen according to the number of data, models 1, 3, 5, and 7 are chosen which have only two blocks for feature extraction. At first, the resolution of 50 by 50 was chosen for this experiment, but after that, the resolution of 100 by 100 also has been tried. 80% of the images of each class are selected for the training set and the others for the validation set. The optimizer and initial learning rate are the same as the previous experiment, and the models were trained for 100 epochs.

#### Discussion

The evaluation results are shown in the Table 2. The F1-score, recall, and precision of the models on each class are reported (BLB: Bacterial Leaf Blight, BS: Brown Spot, LS: Leaf Smut). Although models 1, 3, and 7 have achieved similar average F1-score, model 3 has the highest average precision compared to all the models. In this experiment, the CNN-based model (model1) gained the highest convergence score while having a similar average recall and F1-score with model 3, a ViT based model. Although the work done by^10^ has achieved the highest F1-score on class BLB, but the accuracy on the class LS is pretty low and all the models outperform this work in terms of the average F1-score. All the models also have been trained with the resolution of 100 by 100, but no improvement was observed in any of those four cases. Because of that, it seemed unnecessary to try the resolution of 200 by 200.

### Experiment on plant village

In this paper, the RGB version of images has been used because the classification results could reach higher accuracy in comparison with the grayscale images^12^. Moreover, applying the RGB version of images helps specify different kinds of diseases. Thanks to the hardware advancement, the computation costs of classification with the RGB and grayscale images are almost the same. The Plant Village dataset has more images and classes in comparison with WRCD and RLDD datasets. Because of that, models 2, 4, 6, and 8 have been chosen for this experiment which have more complexity than models 1, 3, 5, and 7. In addition to that, the resolution 200 by 200 were chosen for these models. The number of neurons in the two fully connected layers before the final classifier was increased up to 200. Eighty percent of the images from each class are selected as the training set, and the rest are used for validation. Same as the previous experiments, the optimizer was AdamW, and all the models have been trained for 100 epochs with an initial learning rate of 0.001.

#### Discussion

The evaluation results of the trained models are reported in Table 3. Model 4, which only consists of the transformer blocks, has gained the highest f1-score, recall, and precision. This model outperforms not only model 2, model 6, and model 8 but also AlexNet and GoogleNet, which were trained from scratch in the baseline^12^. Model 4 has far fewer trainable parameters (less than 1 million) in comparison with GoogleNet (23 million) and AlexNet (62 million). When AlexNet and GoogleNet were trained with transfer learning, the reported precision, recall, and F1-score improved and were higher than all the other models. Since the proposed models are hand-designed, it will be computationally costly to train these models on ImageNet to use the initial weights for transfer learning. In this experiment, Model 6 has obtained better accuracy compared to Model 8. This indicates that using the transformer blocks after the CNN blocks is better than using the transformer blocks at the earlier layers. Both models have obtained better accuracy than Model 2, which only has CNN blocks. Similar to the previous experiment, Model 4 has the highest convergence score compared to other models. Model 6 converges just a bit faster than Model 8, while Model 2 converges slower than all other models.

### Prediction speed test

**Figure 6:**
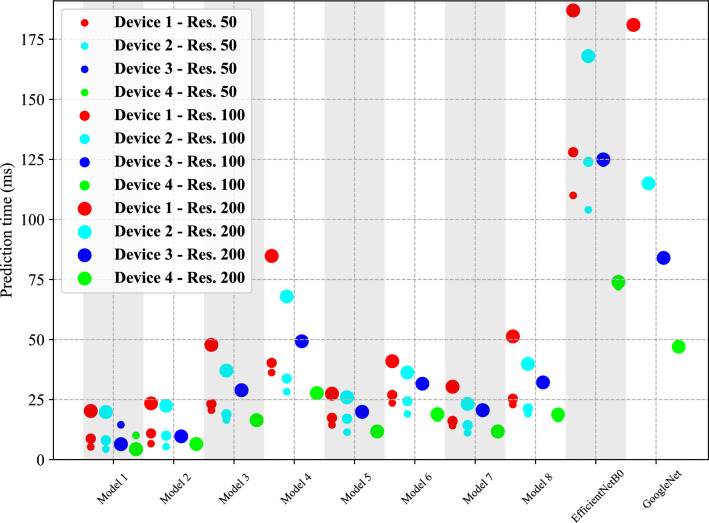
Prediction time of the proposed models with different image input resolution. The code of this paper is available at https://github.com/yasaminborhani/PlantDiseaseClassification.

An experiment has been designed to measure the prediction speed of the proposed models. A sample image was randomly chosen and resized to match the required input resolution of each model. Each model had to then make 1000 predictions based on this sample, and the time for this prediction was measured and averaged. It should be noted that since GoogleNet did not support input resolutions less than 256 by 256, it was examined only with this resolution. All the other models were tested with the resolutions: 50 by 50, 100 by 100, and 200 by 200. The experiment was conducted on four devices; CPU accelerators powered two, and GPU powered the other two. Table 4 shows the specifications for each device. The results of this experiment are demonstrated in Figure 6. At the low resolution , the CPU accelerated devices had less prediction speed compared to device 3, which had GPU. Except for the EfficientNetB0 which device 4 was significantly faster than the others, in the other cases, its prediction speed is almost equal to device 2, which is a CPU accelerated device. At the medium resolution , the GPU accelerated devices are faster than CPU accelerated devices when the models do not have attention blocks (model 1, model 2, and EfficientNetB0), but device 3 was slower than CPU accelerated devices in the models with attention blocks (models 3, 4, 5, 6, 7, 8). The device 4 was the fastest device at this resolution. For the highest resolution, GPUs were significantly faster than CPUs in all cases.

In all resolutions, the purely convolutional-based models (Model 1 and Model 2) were faster than the models with attention blocks. The strictly attention-based models were the slowest compared to all the other hand-designed models. The hybrid models, which have both convolutional and attention blocks, inherited the speed from convolutional layers and are faster compared to purely attention-based models. Whether the convolutional blocks come first (Models 5 and 6) or the network starts feature extraction with the attention blocks (Models 7 and 8), the speed pattern is the same. All the hand-designed models are faster than EfficientNetB0 and GoogleNet on all devices.

## Conclusion

In this paper, a simplified version of the novel Vision Transformer was compared with a convolutional-based architecture with similar complexity. In addition, the hybrid models which included the combination of the CNN and ViT were studied. These networks were employed to solve classification tasks on a small dataset (Rice Leaf Disease Dataset), medium dataset (Wheat Rust Classification Dataset), and large scale dataset (Plant Village). The main challenges with the RLDD dataset were the less number of samples and the similarities between the two given classes. The WRCD dataset had more real scenario images with respect to the two other datasets. The Plant Village was a complex dataset with the skewed classes problem. In all cases, when the models are trained from scratch, the ViT based model not only achieved more accurate performance compared to the CNN or hybrid models, but also it obtained a comparable accuracy with the results reported in the literature. Moreover, the proposed ViT model had considerably lower parameters than the works done in the reviewed papers. However, the attention blocks utilized in this paper were slower than the implemented convolutional blocks on all devices. Combining the attention blocks with convolutional blocks helps the models to predict faster than the ViT-based models while having higher accuracy than the CNN-based models (Plant Village and WRCD experiments). It was also observed that the order of this combination does not significantly affect the prediction speed, but using attention blocks followed by convolutional blocks led to slightly better accuracy (WRCD and Rice Leaf Disease Dataset).

For future work, in order to move toward an automatic disease detection system, object localization networks are required to locate the plant leaves in a wide image, and multi-label classification needs to be solved to take multiple diseases into account. In addition to finding a network structure for object localization and multi-label classification, collecting a dataset with appropriate labels for this problem can be noted as future work of this paper.
